# The Human Centromedial Amygdala Contributes to Negative Prediction Error Signaling during Appetitive and Aversive Pavlovian Gustatory Learning

**DOI:** 10.1523/JNEUROSCI.0926-22.2023

**Published:** 2023-04-26

**Authors:** Emilia Kolada, Krzysztof Bielski, Mateusz Wilk, Krystyna Rymarczyk, Piotr Bogorodzki, Paweł Kazulo, Bartosz Kossowski, Marek Wypych, Artur Marchewka, Leszek Kaczmarek, Ewelina Knapska, Iwona Szatkowska

**Affiliations:** ^1^Neurobiology of Emotions Laboratory, Centre of Excellence for Neural Plasticity and Brain Disorders (BrainCity), Nencki Institute of Experimental Biology, Polish Academy of Sciences, 02-093 Warsaw, Poland; ^2^Behavioral Neuroscience Laboratory, Department of Biological Psychology, SWPS University, 03-815 Warsaw, Poland; ^3^Institute of Radioelectronics and Multimedia Technology, Warsaw University of Technology, 00-665 Warsaw, Poland; ^4^Laboratory of Brain Imaging, Nencki Institute of Experimental Biology, Polish Academy of Sciences, 02-093 Warsaw, Poland; ^5^Laboratory of Neurobiology, Centre of Excellence for Neural Plasticity and Brain Disorders (BrainCity), Nencki Institute of Experimental Biology, Polish Academy of Sciences, Warsaw, 02-093, Poland

**Keywords:** centromedial amygdala, extraversion, gustatory learning, neuroticism, pavlovian learning, prediction error

## Abstract

Prediction error (PE) is the mismatch between a prior expectation and reality, and it lies at the core of associative learning about aversive and appetitive stimuli. Human studies on fear learning have linked the amygdala to aversive PEs. In contrast, the relationship between the amygdala and PE in appetitive settings and stimuli, unlike those that induce fear, has received less research attention. Animal studies show that the amygdala is a functionally heterogeneous structure. Nevertheless, the role of the amygdala nuclei in PE signaling remains unknown in humans. To clarify the role of two subdivisions of the human amygdala, the centromedial amygdala (CMA) and basolateral amygdala (BLA), in appetitive and aversive PE signaling, we used gustatory pavlovian learning involving eating-related naturalistic outcomes. Thirty-eight right-handed individuals (19 females) participated in the study. We found that surprise with neutral feedback when a reward is expected triggers activity within the left and right CMA. When an aversive outcome is expected, surprise with neutral feedback triggers activity only within the left CMA. Notably, the BLA was not activated by those conditions. Thus, the CMA engages in negative PE signaling during appetitive and aversive gustatory pavlovian learning, whereas the BLA is not critical for this process. In addition, PE-related activity within the left CMA during aversive learning is negatively correlated with neuroticism and positively correlated with extraversion. The findings indicate the importance of the CMA in gustatory learning when the value of outcomes changes and have implications for understanding psychological conditions that manifest perturbed processing of negative PEs.

**SIGNIFICANCE STATEMENT** A discrepancy between a prediction and an actual outcome (PE) plays a crucial role in learning. Learning improves when an outcome is more significant than expected (positive PE) and worsens when it is smaller than expected (negative PE). We found that the negative PE during appetitive and aversive taste learning is associated with increased activity of the CMA, which suggests that the CMA controls taste learning. Our findings may have implications for understanding psychological states associated with deficient learning from negative PEs, such as obesity and addictive behaviors.

## Introduction

In human studies on neural correlates of pavlovian learning, the amygdala, a key structure processing emotions in the brain, is most commonly considered as a whole. However, animal studies show that the amygdala is a heterogeneous structure. Its various parts, particularly the basolateral amygdala (BLA) and the central nuclei (CA), apparently serve different subsystems and are involved in distinct aspects of the pavlovian learning process. In particular, the BLA forms a conditioned stimulus–unconditioned stimulus association (Keefer and Petrovich, 2017; Sun et al., 2020). In contrast, the CA encodes the motivational significance of stimuli and modulates conditioned responses (Murray, 2007; Averbeck and Costa, 2017; Fadok et al., 2018; Warlow and Berridge, 2021).

There is strong evidence that pavlovian learning depends on the predictive relationship between events, not just their temporal contiguity (Rescorla and Wagner, 1972). Hence, prediction errors (PEs), defined by the discrepancy between actual and expected outcomes, lie at the core of associative learning. PEs are necessary to increase attention and update the motivational significance of stimuli when contingencies change (Bissonette and Roesch, 2016). PEs can be positive when the outcome is greater than expected or negative when the outcome is smaller than expected. Positive PEs increase, whereas negative PEs decrease, the motivational significance of stimuli and their associative strength (Rescorla and Wagner, 1972). Furthermore, PEs are related to either appetitive or aversive outcome valence (Iordanova et al., 2021). The brain correlates of the two dimensions of PEs, outcome direction (positive or negative PEs) and outcome valence (appetitive or aversive PEs), are poorly understood.

The amygdala has been linked predominantly to aversive PE by studies that used fear learning. Rodent studies implicated the BLA in the process (for review, see Iordanova et al., 2021). The role of the amygdala in PE signaling in appetitive settings has received less research attention. Nevertheless, a rodent experiment showed that the intact CA is crucial for learning enhancement caused by food reward omission (Holland and Gallagher, 2006). Furthermore, the CA projection to the substantia nigra is implicated in PE signaling while omitting expected food rewards (Lee et al., 2010). These findings of animal studies indicate the role of the CA in negative PE signaling during appetitive learning.

To address the open question about the human amygdala correlates of positive and negative PEs during appetitive and aversive pavlovian gustatory learning, we probed responses of the BLA and the centromedial amygdala (CMA) complex including CA in an fMRI experiment. We analyzed the CMA activity because the low spatial resolution of an fMRI method makes it difficult to demonstrate CA activity in human fMRI studies. Based on animal research, we predicted that the BLA would be associated with positive and negative PE signaling during aversive learning, whereas the CMA would be involved in negative PE signaling during appetitive learning. In addition, we explored the effects of body mass index (BMI), neuroticism, and extraversion on BLA and CMA activity as those factors might modulate amygdala activity during pavlovian learning (Klucken et al., 2019), PE signaling (Smillie et al., 2019), and reward processing (Stice and Yokum, 2016). Unlike previous studies, we used dynamic and multimodal natural stimuli comprising gustatory and social components. The stimuli consisted of small portions of liquid (appetitive, aversive, or neutral) and a short video showing a person drinking this liquid and reacting to its taste. The rationale for using more ecologically relevant stimuli was twofold. First, dynamic, primary reinforcers evoke more robust PE-related activity of the amygdala than static, secondary reinforcers such as monetary gains (Metereau and Dreher, 2013). Adding dynamic social context to appetitive and aversive liquid consumption further increases amygdala involvement (Rymarczyk et al., 2018, 2019). Second, using gustatory stimuli in both appetitive and aversive settings enabled us to compare the amygdala activations induced by stimuli of the same modality. Such comparisons are not possible for rewards and fear-inducing stimuli used in several previous studies.

## Materials and Methods

### Subjects

Thirty-eight right-handed individuals (19 females; average age, 25 years ± 2.85 SD, range 21–33 years) participated in the study. In accordance with the study eligibility criteria, all subjects declared normal or corrected-to-normal vision and no diagnosed neurologic or psychiatric disorders, brain damage, epilepsy, diabetes, or claustrophobia. Because of familiarity with the pavlovian learning process, holders of psychology degrees and psychology students (third year or higher) were not allowed to participate. Because the reinforcing stimuli used in the experiment were food related, and satiety might affect the appetitive value of gustatory stimuli (Berridge, 2012), subjects were asked to fast before the experiment for at least 4 h. The experimental procedure was approved by the Human Ethics Committee of the SWPS University of Social Sciences and Humanities in Warsaw, Poland. All participants gave written informed consent before the study and received financial compensation (100 Polish zloty, ∼25 euros) after the study.

### Experimental design

#### Before the experiment

On the day of the experiment, the subjects underwent psychological assessment. Neuroticism and extraversion were measured using the Neuroticism, Extraversion, Openness (NEO) Five-Factor Inventory (NEO-FFI; Costa and McCrae, 1992; Zawadzki et al., 1998). Next, subjects were asked to choose the most palatable liquid. The selection of sweet drinks involved chocolate milk, orange juice, apple juice, strawberry juice, and black currant juice. Finally, the participants were given small amounts of three types of liquids that were used in the experiment: a selected sweet drink, a salty solution, and a neutral solution. The participants were asked to try them and rate their pleasantness using an 11-point Likert scale ranging from −5 (very unpleasant) to +5 (very pleasant).

#### Pavlovian learning task

**Figure 1.**
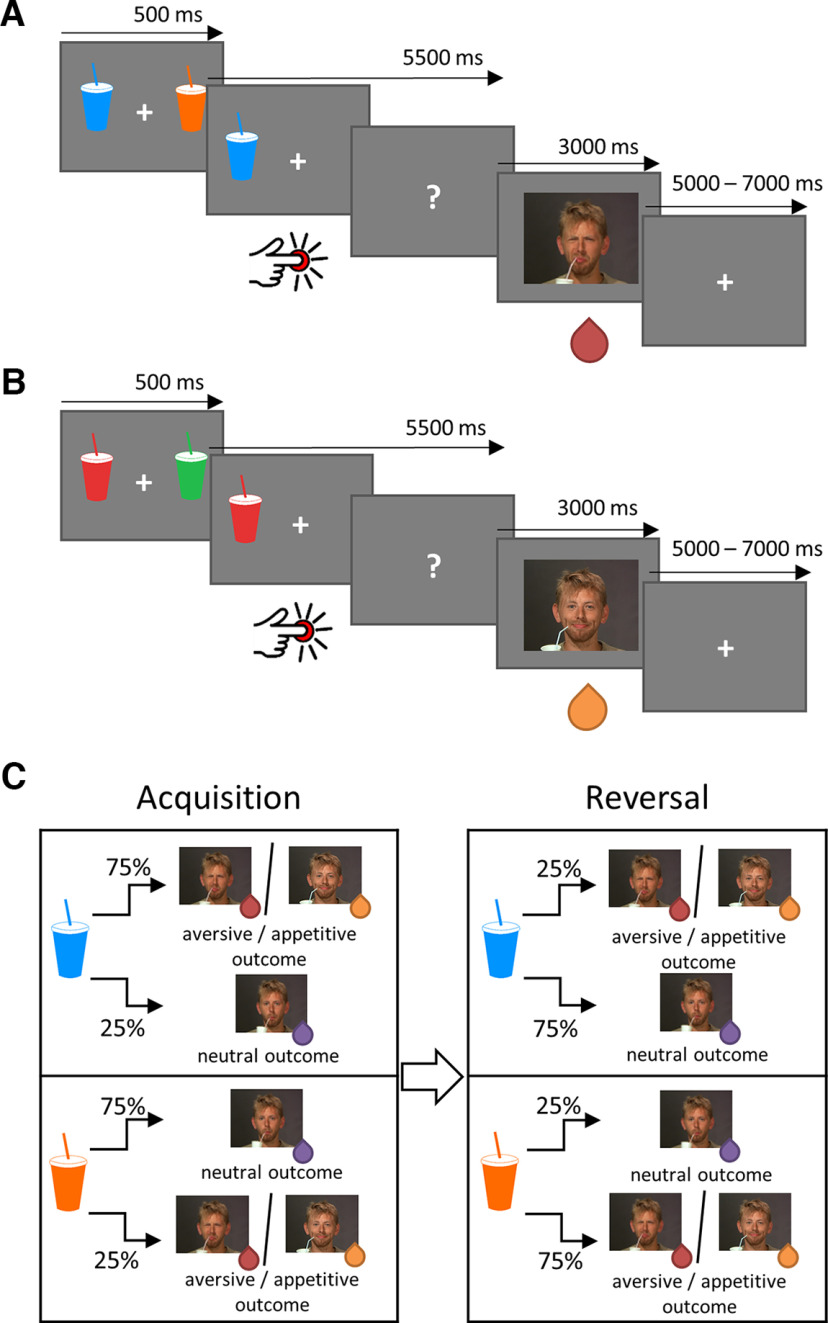
Experimental paradigm. ***A***, ***B***, A course of a single trial in aversive (***A***) and appetitive (***B***) sessions. A trial started with a presentation of two cues, one of which disappeared after 500 ms. A participant had to decide, by pressing a button, what would follow the remaining cue—an aversive/appetitive outcome or a neutral outcome. After a button was pressed, before the outcome was displayed, a question mark was presented. In the reinforcement phase, the liquid was administered, and the video was presented. A fixation cross indicated the end of a trial. Intertrial intervals varied between 5000 and 7000 ms. ***C***, Reinforcement scheme. One of two cues was associated with a higher probability (75%) of an aversive/appetitive outcome and a lower probability (25%) of a neutral outcome. Conversely, the other cue was predictive of a neutral outcome more often (75% probability) than an aversive/appetitive outcome (25% probability). Five successive correct responses resulted in reversal of this association, which could occur in one of the next three trials. Pictures of the reinforcing stimulus in the figure used with permission of the authors of the video (Jabbi et al., 2007).

The experimental procedure was written and presented with Presentation software (Neurobehavioral Systems). The experiment consisted of four sessions; two were appetitive (palatable and neutral reinforcement) and two aversive (unpalatable and neutral reinforcement). To counterbalance the order, half the participants started with an appetitive session, and the other half started with an aversive session. One session consisted of 40 trials. Each trial began with a presentation of two cues, one on the right side of the screen and one on the left. After 500 ms, one of the cues disappeared, and the other one remained on the screen as long as a participant pressed the left or right button but no longer than 5.5 s. The task was to predict whether the remaining cue was associated with a palatable/unpalatable or neutral liquid and indicate it by pressing one of the buttons corresponding to each of the possible outcomes. After a button was pressed, a question mark appeared, indicating anticipation of an outcome of a trial. In the reinforcement phase, the liquid was administered, and the video was presented (Fig. 1*A*,*B*). One cue in a pair was followed by an affective (appetitive or aversive) outcome for 75% of the trials and a neutral outcome for 25% of the trials [a high probability (HP) affective cue], and the other cue was followed differently; for 25% of the occasions the outcome was appetitive/aversive, and for 75% of the occasions it was neutral (HP neutral cue). This association was reversed every time a subject responded correctly in five consecutive trials (i.e., consistent with a higher probability of the outcome; Fig. 1*C*). Reversal occurred in one of three trials after five correct responses to ensure that the onset of the reversal was not fully predictable by subjects (Prévost et al., 2013). The number of trials starting with HP affective cues was equal to the number of trials starting with HP neutral cues.

Participants were instructed to try to predict the outcome based on a cue, given that one cue was more predictive of an affective outcome and the other cue of a neutral outcome. Participants were also informed about the possible reversal of the cue–outcome association, but the rule governing this change was not explained.

### Stimuli

Visual stimuli (pictures of colored cups) were used as cues. In each of four sessions, two cups of different colors were presented, and for each session, a new set of colors was used. One color was associated with a higher probability of an affective outcome, and the other color was associated with a higher probability of a neutral outcome. We used green and red cups in one appetitive session and brown and pink cups in another appetitive session. For aversive learning, we used blue and orange cups in one session and violet and yellow cups in another session. The presentation side of HP neutral and HP affective cues on the screen was counterbalanced.

Reinforcers were a gustatory component (palatable, unpalatable, or neutral liquid) and a visual component (a short video showing a person's facial expression while drinking). The liquids used in the experiment were sweet (chocolate milk or fruit juice; see above, Before the experiment), salty (0.4 m NaCl), and neutral (25 mm KCl, 2 mm NaHCO_3_). In each trial, participants received 0.5 ml of a liquid via Teflon tubes attached to semiautomatic programmable syringe pumps. Concurrent with liquid administration, a 3 s video of a person expressing pleasure, disgust, or no particular emotion while drinking palatable, unpalatable, or neutral beverages was presented. The videos were used with permission of Jabbi et al. (2007), who described and used the stimuli to investigate neural processes underlying the observation of taste-related emotional responses in other people. Liquids and videos were congruent with each other in terms of affect and were combined to invoke strong and highly positive (appetitive), negative (aversive), or neutral states in subjects.

### Computational model

The estimates of prediction errors were calculated for each subject using their responses in the task. We implemented a standard Rescorla–Wagner (RW) model of learning (Rescorla and Wagner, 1972), which defines a prediction error (δ) as the difference between the actual outcome *R* and the expected value *V* in a given trial *t* as follows: $\delta_{t}=R_{t}-V_{t}.$

The expected value in the next trial is the sum of the expected value and the prediction error weighted by a learning rate (α) as follows: $V_{t+1}=V_{t}+\alpha \delta_{t}.$

In each trial, the value of *V* was associated with the individual expectation that the presented cue would be followed by the outcome that was initially more strongly linked with it by the current session design. The concurrence of this type was coded as *R* = 1, and its violation was coded as *R* = 0. The initial expected value (*V_0_*) was set to 0.5, and individual learning rates were constant throughout a single run. Subsequently, we transformed the expected values $V_{t}$ to obtain the measurements of the anticipation of the non-neutral liquid under the presented cue. If the cue presented in trial *t* was initially more strongly linked with the non-neutral liquid by the current session design, then $V\prime_{t}=V_{t},$ otherwise, $V\prime_{t}=1-V_{t}.$ We also expressed the new prediction errors accordingly as $\delta^{\prime }{}_{t}=R^{\prime }{}_{t}-V^{\prime }{}_{t},$ where $R\prime_{t}=1$ for trials with the non-neutral liquid as the outcome and $R\prime_{t}=0$ otherwise. The parameters were estimated using a Bayesian inference framework as implemented in the Hierarchical Gaussian Filter (HGF) Toolbox (Frässle et al., 2021).

To confirm that the model we used to estimate PEs was the best choice, two other models were fitted to behavioral data. First, the temporal difference (TD) learning model (Sutton, 1988; Sutton and Barto, 1990) was applied because it is a dynamical extension of the RW model. Second, the HGF model (Mathys et al., 2014) was selected as a Bayesian alternative for reinforcement learning. For each model, the Akaike information criterion (Akaike, 1974) was calculated, and the values were compared with Friedman's rank test, which showed a significant difference between model evidence (appetitive and aversive task, χ^2^(2) = 28.0, *p* < 0.001). *Post hoc* analysis with the Wilcoxon test confirmed that the RW model performed better than the TD model (in both appetitive and aversive runs at p* < 0.001) and the HGF model (in appetitive runs at p* < 0.046 and aversive runs at p* < 0.032). The *p* values were Bonferroni corrected (p*).

### MRI data acquisition and preprocessing

#### Acquisition

Data were acquired using a Siemens Trio 3T MRI scanner equipped with a 32-channel head coil. We collected 405 whole-brain functional images per run using the T2*-sensitive multiband accelerated echoplanar imaging (EPI) sequence with the following parameters: 2.2 mm isotropic resolution, TR = 1.5, TE = 0.029, flip angle = 70 degrees, multiband factor = 3, FOV = 211.2 mm × 211.2 mm, matrix = 96 × 96 × 66). A high-resolution T1-weighted structural image (1 mm isotropic resolution, TR = 2.53 s, TE = 0.003 s, flip angle = 7 degrees, FOV = 256 mm, matrix = 256 × 256 × 176) was acquired for the purpose of normalizing the functional data.

#### Preprocessing

Data were collected from 38 participants. One subject was excluded from the analysis because of excessive head movements. In another six subjects, the analyzed data were incomplete (including three sessions or fewer of four sessions) because of scanner failure and other technical issues (four subjects), head motion (one subject), and discomfort related to the experimental procedure (one subject).

Data were preprocessed and analyzed with SPM12 (Wellcome Centre for Human Neuroimaging, University College, London; https://www.fil.ion.ucl.ac.uk/spm/) using default settings. To compensate for the head movement, all volumes were realigned to the first image using rigid body transformation. Additional movement outliers were identified with ARtifact Detection Tools (ART). Outliers were detected using composite motion measure and a threshold equal to half a voxel size, or 1.1 mm. A session was included in the analysis if the number of outliers was not >10% of all the volumes in this session. The T1-weighted structural image was coregistered to the mean functional image, and all EPI volumes were subsequently normalized to standard stereotactic Montreal Neurologic Institute (MNI) space using a unified segmentation procedure, which involves segmentation using tissue probability maps, bias correction, and spatial normalization. Normalized images were sliced to achieve voxel dimensions of 2 × 2 × 2 mm. Finally, a Gaussian kernel with an FWHM of 4 mm was applied to smooth the functional images.

### Statistical analysis

#### Analysis outline

Behavioral data analysis involved assessment of pleasantness ratings and test performance. Pleasantness ratings were inspected to specify valence of each gustatory component of stimuli. Next, we calculated the ratios of correct responses for each condition and compared them with the value of 0.5 to verify whether the performance was above the chance level. Ratios of correct responses were also correlated with the scores on neuroticism and extraversion to check whether there is a relationship between task performance and personality traits.

The MRI data were modeled using the general linear model (GLM) framework. Next, we proceeded with the region of interest (ROI) approach to answer more specific questions about the engagement of amygdala subdivisions in associative learning. Four regions were chosen, the left and right BLA and CMA (see Fig. 4*A*). The masks of these subdivisions were calculated with a functional parcellation method based on blood oxygen level-dependent (BOLD) signal dynamics and recurrence quantification analysis (RQA; Bielski et al., 2021). The masks were first applied in a small volume correction (SVC) procedure to detect any suprathreshold clusters of activity within the amygdala subregions, providing information about their size and location. The subsequent parametric modulation analysis of ROIs used the mean signal in each amygdala subdivision to determine the regions with significantly inflated activity, thus revealing those subdivisions that are recruited while processing a particular PE type. This was repeated with the amygdala mask based on structural connections (Bach et al., 2011) to confirm our results. Next, after identifying active subdivisions, we conducted 2 × 2 repeated measures ANOVAs with the factors PE type (positive, negative) and learning type (appetitive, aversive), separately for each activated amygdala subdivision. Also, we performed effective connectivity analysis using structural equation modeling (Dorfman et al., 2021; Ramsey et al., 2010). Structural equation modeling is a statistical method to evaluate the consequences of a set of causal assumptions (that correspond to different structural equation models) and to measure how well they fit the data. In the rest of the article, we refer to causal relations between pairs of regions favored by this analysis as “functional coupling” or “effective connectivity” (Dorfman et al., 2021). As the interaction between the amygdala and orbitofrontal cortex (OFC) seems to be crucial for flexible stimulus–reinforcement learning and value updating (Gottfried et al., 2003; Pujara et al., 2022), our models included activated amygdala subdivisions and OFC areas. Finally, a correlation analysis was performed to establish the relationship between the previously observed brain activity and some individual factors like BMI and personality traits.

#### Behavioral measures

Pleasantness ratings for each liquid were compared with nonparametric tests because of violation of distribution normality assumption. To analyze the differences between the liquid ratings (separately before and after the experimental measurements), we used the Friedman test and *post hoc* Wilcoxon tests with Bonferroni correction. The stability of ratings across time was assessed with Wilcoxon signed rank tests. The ratio of correct choices was tested against chance level (0.5) with a one-sample *t* test, and correlation between task performance and personality scores was measured with the Pearson correlation coefficient. Statistics were calculated using Python 3.6 and the SciPy and scikit-posthocs packages.

#### fMRI data

For each subject, we computed the first-level GLM, which comprised four conditions of interest—a reinforcing event preceded by a cue with a high probability of an appetitive outcome, a reinforcing event preceded by a cue with a high probability of a neutral outcome in the appetitive run, a reinforcing event preceded by a cue with a high probability of an aversive outcome, and a reinforcing event preceded by a cue with a high probability of a neutral outcome in the aversive run. The duration of these events was set to 3 s (equal to the length of a video stimulus). Next, we applied the parametric modulation technique. Each of the above conditions was modulated with PE estimates, which allowed us to infer the neural representation of negative PEs in appetitive sessions, positive PE in appetitive sessions, negative PE in aversive sessions, and positive PE in aversive sessions. Other regressors of no interest involved the occurrence of cues and their parametric modulator (expected values), the onset of both cues, button press, six head movement regressors, and movement outliers identified with ART. All the missed trials were modeled as a separate regressor. Low-signal frequencies were removed using the default high-pass filter of 128 s.

#### ROI analysis

One-sample *t* test with *p* < 0.05 FWE-corrected (with an initial cluster-defining threshold of *p* < 0.001 uncorrected) was used in the SVC approach. We combined all four ROIs into one mask to calculate the *p* values and thus avoided the issue of testing multiple independent regions. In the ROI analysis, for each of four masks, the extracted beta estimates for each subject were averaged across all of the voxels in a mask and then subjected to a one-sample *t* test. The reported *p* values were Bonferroni corrected to account for the number of ROIs (p*). As the choice of a method for amygdala parcellation might have an impact on the final findings (Kolada et al., 2017), we then verified our results using a structural parcellation method that differentiated two amygdala subdivisions based on anatomic connections (Bach et al., 2011). The computations were performed in MATLAB R2022b (MathWorks) software.

#### Effective connectivity analysis

We have included the CMA, posterior medial OFC (mOFC) and lateral OFC (lOFC) in the structural equation modeling models. To create models, we have taken into account following findings: (1) It was shown that the OFC, and no other prefrontal regions, plays a necessary role in flexible stimulus–reinforcement learning in humans (Tsuchida et al., 2010); (2) one of the few studies that examined structural connectivity between amygdala subdivisions and OFC in humans found that the CMA exhibited a strong connection to the OFC (Abivardi and Bach, 2017), and more recent human findings suggest that the CMA may connect preferentially with medial and posterior OFC (Matyi and Spielberg, 2021); (3) the medial OFC and lateral OFC are massively interconnected and functionally interact (Nallapu and Alexandre, 2019); and (4) lateral OFC was shown to be involved in the negative PE signaling in human subjects (Nahum et al., 2011; Mollick et al., 2021).

Amos (IBM SPSS Statistics, version 26.0 software) was used for structural equation modeling path analysis. An anatomic model consisted of three regions: CMA (Bielski et al., 2021), posterior mOFC, and lOFC (Henssen et al., 2016) separately for left and right hemisphere. Percentage signal change for either appetitive or aversive sessions were calculated for anatomic model ROIs using home-written MATLAB script and subsequently used as input for structural equation modeling analysis. A sample size (37 for negative PEs in appetitive sessions and 36 for negative PEs in aversive sessions) was checked (Bentler and Chou, 1987), to fulfill the rule that states that a sample size should be at least equal to a number of parameters times five criteria. Model fit was tested using the chi-square test with 0.05 significance level.

#### Correlation analysis

The mean beta values that were significantly elevated in the ROI analysis were passed to a correlation analysis. Specifically, the strength of a relationship between brain activity and (1) BMI, (2) neuroticism, and (3) extraversion scores were measured by means of the Pearson correlation. The coefficients and statistical thresholds were calculated in MATLAB software. All results with the *p* value below 0.05 are reported below.

## Results

### Behavioral results

#### Pleasantness ratings of liquids

**Figure 2.**
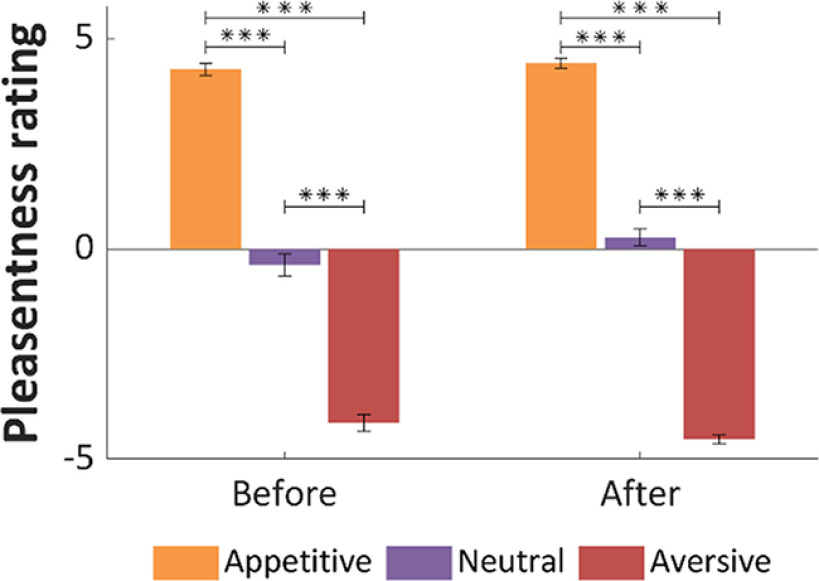
Mean pleasantness ratings before and after the experiment for three types of liquids, appetitive, neutral, and aversive. Error bars indicate SEM; ***p < 0.001.

Subjects rated the gustatory stimuli before and after fMRI sessions. Differences between ratings of neutral, appetitive, and aversive liquids were significant at both measurements, before the experiment (χ^2^(2) = 69.524, *p* < 0.001) and after the experiment (χ*^2^*(2) = 71.51, *p* < 0.001). The *post hoc* Wilcoxon test showed differences in each pair of ratings that was compared (p* < 0.001 in all tests; Fig. 2). Furthermore, we tested whether the ratings were constant across time. The neutral stimulus was rated more pleasant (*T* = 19.5, *p* = 0.008), and the aversive stimulus was rated more unpleasant (*T* = 16, *p* = 0.036) after the experiment, whereas the ratings of appetitive liquids did not differ significantly between the measurements. Figure 2 presents mean pleasantness ratings before and after the experiment.

#### Task performance

**Figure 3.**
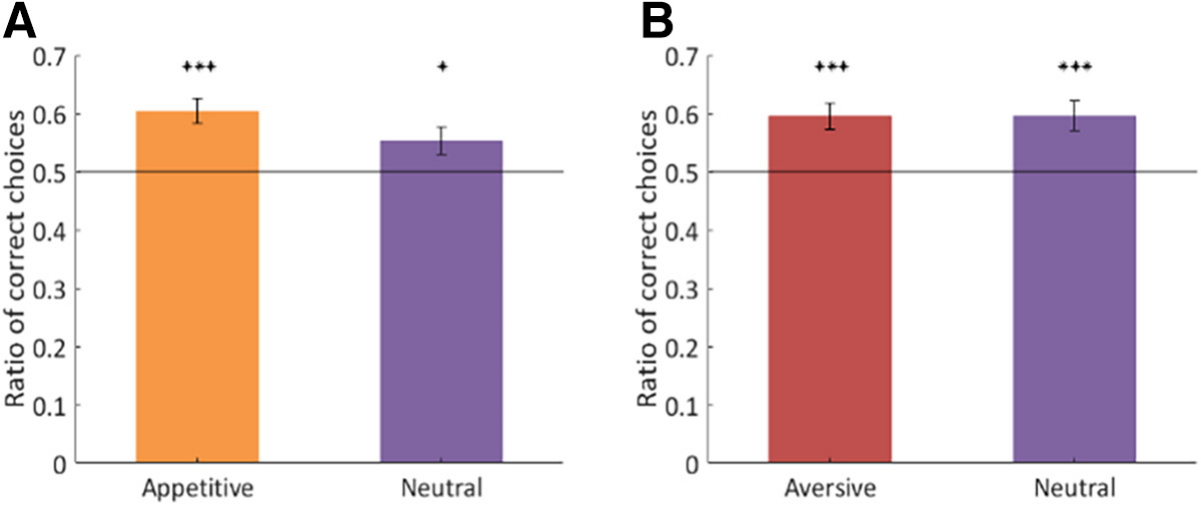
***A***, Ratio of correct responses averaged across appetitive sessions and subjects. ***B***, Ratio of correct responses averaged across aversive sessions and subjects. Error bars indicate SEM. Horizontal line represents chance level. The ratios were tested against the chance level of 0.5 with one sample *t* tests; *p < 0.05, ***p < 0.001.

As a measure of learning, we calculated average ratios of correct responses for each of four conditions separately. The results of a one-sample *t* test showed that the mean ratios of correct choices for appetitive (mean = 0.6037, *t*_(36)_ = 4.94, *p* < 0.001) and neutral (mean = 0.5528, *t*_(36)_ = 2.26, *p* < 0.05) conditions in appetitive sessions were significantly above the chance level. Likewise, in the aversive sessions the accuracy for aversive (mean = 0.5954, *t*_(35)_ = 4.35, *p* < 0.001) and neutral (mean = 0.5959, *t*_(35)_ = 3.73, *p* < 0.001) conditions differed significantly from 0.5. The results are presented in Figure 3. We also tested whether the ratio of correct responses varied significantly between the conditions. A paired *t* test revealed that the percentage of correct choices was greater for the appetitive condition (*t*_(36)_ = 2.7, *p* < 0.05) than for the neutral condition in appetitive sessions. No such difference was observed for aversive and neutral conditions in aversive sessions.

Finally, we did not find any correlation between task performance and personality traits that would achieve the threshold for statistical significance.

### fMRI results

**Figure 4.**
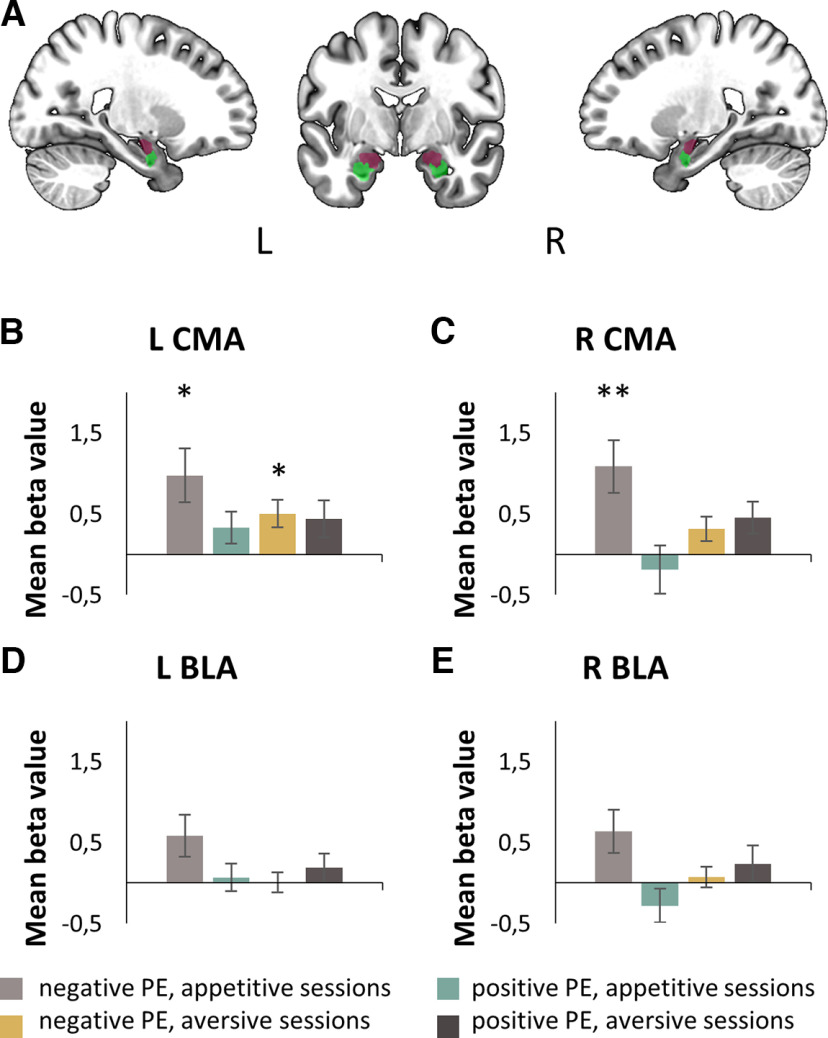
Results of parametric modulation analysis in the amygdala subdivisions. ***A***, ROIs defined based on resting-state fMRI and RQA analysis. Left and right CMA in purple, left and right BLA in green. ***B***, Mean beta values for the left CMA. ***C***, Mean beta values for the right CMA. ***D***, Mean beta values for the left BLA. ***E***, Mean beta values for the right BLA. Colors in the bar graph represent the following: light gray, negative PE signal in appetitive sessions; green, positive PE signal in appetitive sessions; yellow, negative PE signal in aversive sessions; dark gray, positive PE in aversive sessions. Mean parameter estimates were tested with a one-sample *t* test, Bonferroni corrected, *p < 0.05, **p < 0.01. Error bars indicate SEM.

The SVC approach revealed significant modulation of BOLD by PEs during reinforcing events preceded by either HP appetitive or aversive cues but not HP neutral cues (Table 1). Parametric modulation analysis of amygdala ROIs confirmed the results obtained by correcting for the small volume and involvement of the amygdala in associative learning. We found a positive correlation of amygdala activity with PE values during reinforcement trials preceded by an HP cue for appetitive and aversive reinforcements. Specifically, the left CMA (*t*_(36)_ = 2.93, p* < 0.05) and right CMA (*t*_(36)_ = 3.32, p* < 0.01) were active when the PE was related to the expectation of an appetitive outcome (negative PE in appetitive sessions; Fig. 4*B*,*C*), and only the left CMA subdivision of the amygdala (*t*_(35)_ = 2.95, *p* < 0.05) was active when the PE was related to the expectation of an aversive outcome (negative PE in aversive sessions; Fig. 4*B*). The same pattern of activation was observed when alternative masks based on anatomic connections were used (Bach et al., 2011). Negative PEs in appetitive sessions triggered activity in the left (p* = 0.013) and right (p* = 0.008) superficial subdivision of the amygdala (corresponding to the CMA), whereas negative PEs in aversive sessions triggered activity in the left superficial subdivision of the amygdala alone (p* = 0.009).

**Table 1:** PE-related activity in the amygdala after small volume correction

Region	MNI coordinates			Cluster size	Cluster	Peak	*T*	*Z*
	*x* (mm)	*y* (mm)	*z* (mm)	*k*	*p*_FWE_	*p*_FWE_		
Negative PEs in appetitive sessions								
L CMA	−20	−10	−14	13	0.006	0.013	4.43	3.93
R CMA	22	−8	−14	15	0.005	0.018	4.29	3.83
L CMA	−18	−2	−18	2	0.065	0.024	4.18	3.75
Positive PEs in appetitive sessions								
No voxels surviving the threshold								
Negative PEs in aversive sessions								
L CMA	−22	−4	−16	11	0.008	0.027	4.15	3.72
Positive PEs in aversive sessions								
No voxels surviving the threshold								

The cluster-defining threshold was set to *p* < 0.001 uncorrected. L, Left; R, right.

Next, separately for the left and right CMA, we conducted repeated-measures ANOVAs with PE type (positive and negative) and learning type (appetitive and aversive) as factors. In the case of the left CMA, the main effect of PE type reached a trend level (*F*_(1,36)_ = 2.95, *p* = 0.094). Negative PEs triggered higher activity than positive PEs. There was no significant effect of learning type (*p* > 0.4) nor the interaction (*p* > 0.2). For the right CMA, there was a significant main effect of PE type (*F*_(1,36)_ = 5.112, *p* = 0.030). Negative PEs triggered higher activity than positive PEs. There was no significant effect of learning type (*p* > 0.8), but the interaction was statistically significant (*F*_(1,36)_ = 9.244, *p* = 0.004). *Post hoc* comparisons revealed that negative PEs recruited the right CMA only in an appetitive context of learning (*t*_(36)_ = 3.723, *p* = 0.002).

#### Effective connectivity analysis

**Figure 5.**
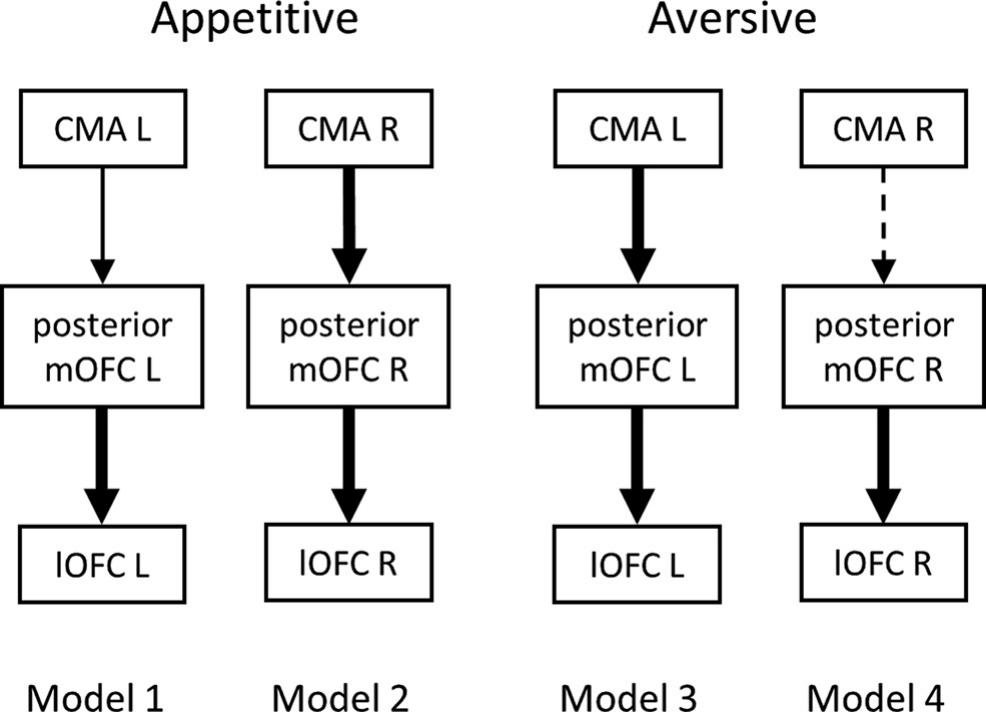
Path diagrams from structural equation modeling analysis based on fMRI data of appetitive and aversive sessions. Region variables are represented as rectangles, and paths between them are represented as arrows. Model fit parameters are presented under corresponding diagrams (there is 1 degree of freedom in every model). Solid thick arrows are paths with coefficients statistically significant at the 0.05 level, solid thin arrow represents a path with coefficient significant at the 0.1 level, and dashed arrow represents a path with coefficient not significant at any level. L, left; R, right.

Effective connectivity between CMA and OFC subregions was investigated for four models presented in Figure 5. Evaluation of model fit proved that the models were not significantly different from the data (model 1, χ^2^ = 1.59, *p* = 0.21; model 2, χ^2^ = 0.60, *p* = 0.44; model 3, χ^2^ = 0.01, *p* = 0.94; model 4, χ^2^ = 0.16, *p* = 0.69). The estimated path coefficients for both sessions are reported in Table 2. Effective connectivity between the right CMA and posterior mOFC during negative reward PE signaling, as well as between the left CMA and posterior mOFC during negative aversive PE signaling were statistically significant. Connectivity between the left CMA and posterior mOFC during negative reward PE signaling reached the trend level.

**Table 2:** Path coefficients resulting from structural equation modeling analysis of left- and right-hemisphere models in appetitive and aversive sessions

Path	Path coefficients in appetitive sessions	*p* Value	Path coefficients in aversive sessions	*p* Value
CMA R→posterior mOFC R	0.29 (0.11)	0.007	0.02 (0.28)	0.931
posterior mOFC R→lOFC	0.66 (0.14)	***	0.51 (0.11)	***
CMA L→posterior mOFC L	0.19 (0.10)	0.065	0.42 (0.19)	0.029
posterior mOFC L→lOFC L	0.71 (0.13)	***	0.51 (0.16)	0.002

***Significant at 0.001 level. SEM is in parentheses. L, Left; R, right.

#### Correlation analysis

In the correlation analysis we considered only the significant results, i.e., the mean β values estimated for the negative PEs for the right CMA in appetitive sessions and for the left CMA in appetitive and aversive sessions (despite the nonsignificant main effect of PE type in this case).

Multiple studies have shown a relationship between neural responses in the amygdala and weight (Boutelle et al., 2015); therefore, we also calculated BMI for each subject. The values of the index ranged from 17.58 to 33.21, with a mean BMI equal to 23.25 (SD = 3.13).

Next, we correlated the mean beta values with personality traits (neuroticism and extraversion) measured with the NEO-FFI questionnaire. Previous studies have shown that higher levels of neuroticism are associated with decreased amygdala activity during appetitive learning (Schweckendiek et al., 2016; Klucken et al., 2019) and increased amygdala activity during fear learning (Hooker et al., 2008). High extraversion was shown to be related to enhanced appetitive PE signaling (Smillie et al., 2019). Higher levels of BMI are associated with greater responsivity of brain regions associated with reward and motivation (Stice and Yokum, 2016) and with deficient learning from negative appetitive PE (Mathar et al., 2017). Given these findings, we tested whether (1) a higher level of neuroticism predicts weaker appetitive PE signaling and enhanced aversive PE signaling in the amygdala; (2) a higher level of extraversion predicts enhanced appetitive PE signaling in the amygdala; and (3) a higher level of BMI predicts weaker negative appetitive PE signaling in the amygdala.

**Figure 6.**
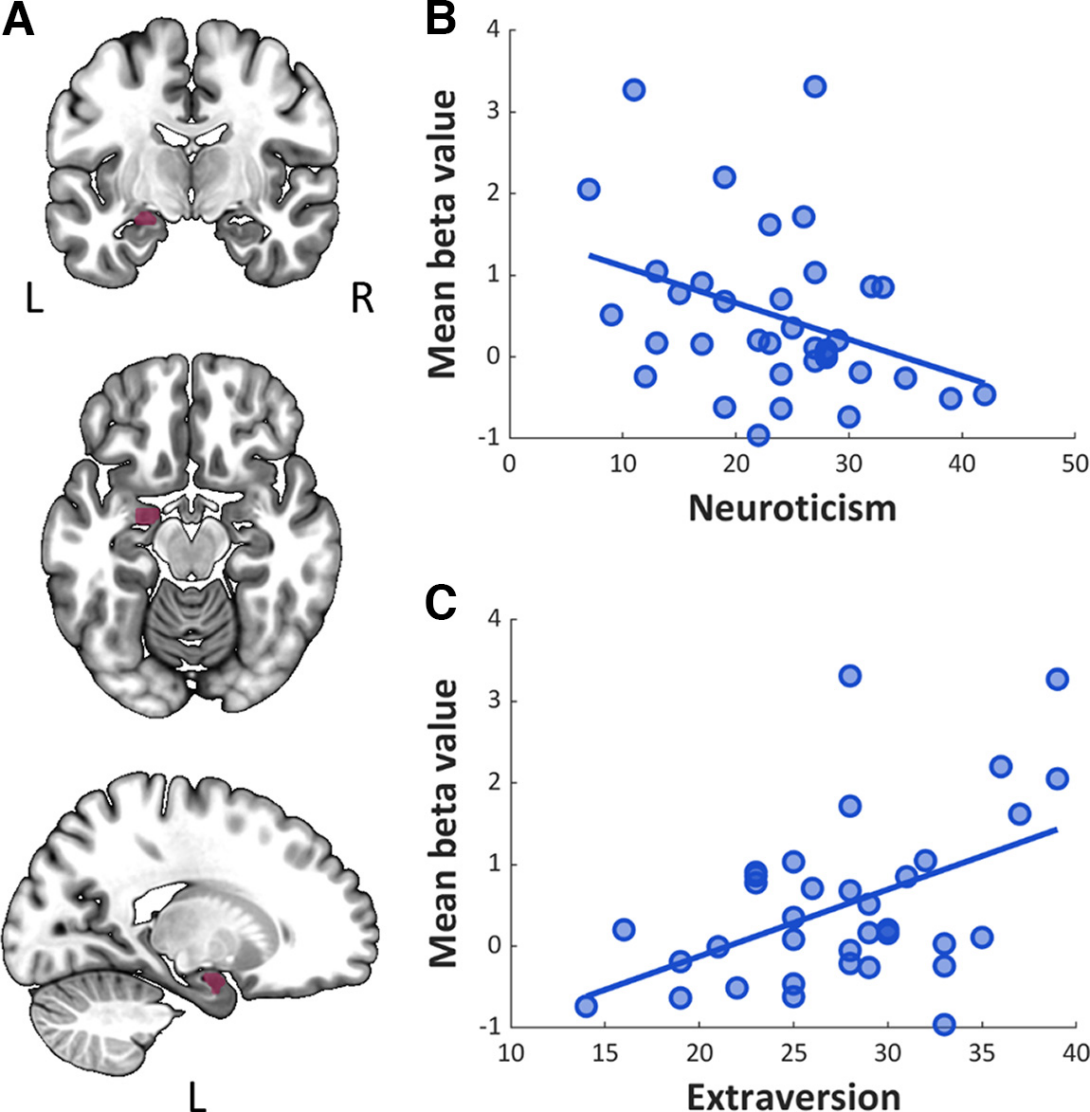
Results of the correlation analysis between personality traits and activity in the left CMA in response to negative PEs in aversive sessions. ***A***, Left CMA. ***B***, Correlation with neuroticism score. ***C***, Correlation with extraversion score. L, left hemisphere; R, right hemisphere.

The PE signal in the left CMA correlated with both neuroticism and extraversion but only in aversive sessions and in the opposite manner (Fig. 6). Specifically, the higher the level of neuroticism, the lower the PE-related activity in the left CMA (*r*_(36)_ = −0.36, *p* = 0.032) and the higher the extraversion level, the higher the CMA activity (*r*_(36)_ = 0.48, *p* = 0.003). The correlation between BMI and negative PE-related neural response in the left and right CMA did not yield any significant results. Moreover, none of the personality traits were correlated with BMI (neuroticism, *r*_(35)_ = −0.09, *p* = 0.6; extraversion, *r*_(35)_ = −0.02, *p* = 0.93).

## Discussion

The study aimed to clarify the role of CMA and BLA subdivisions in appetitive and aversive pavlovian learning involving eating-related naturalistic outcomes. The analyzed measures of learning were PEs, determined based on the Rescorla–Wagner theory, which considers the role of surprise in the acquisition of information and skills. We observed that surprise with neutral feedback in appetitive sessions (i.e., when the PE is related to the lack of expected reward) triggers activity within the right and left CMA. In contrast, surprise with neutral feedback in aversive sessions (when the PE is related to the lack of expected aversive taste) triggers activity only within the left CMA. The results show that the CMA is involved in negative PE signaling during both appetitive and aversive gustatory pavlovian learning and that the BLA is not critical for this process. Repeated-measures ANOVA with PE type (positive, negative) and learning type (appetitive, aversive) as factors confirmed the involvement of the right CMA in negative PE signaling during appetitive learning. It also showed the importance of the left CMA for both appetitive and aversive learning and a tendency of this region toward negative PE signaling. In addition, structural equation modeling pointed to the coupling between the right CMA and OFC during negative appetitive PE signaling, between the left CMA and OFC during negative aversive PE signaling, and, to a lesser extent (only trend level), between the left CMA and OFC during negative appetitive PE signaling.

Work with rodents shows that the central amygdala (CA) is crucial for updating the motivational significance of stimuli and surprise-induced learning enhancement (Holland and Gallagher, 2006). In particular, the CA was shown to be involved in the incremental attentional processing that occurs as a result of a downward shift in reward value (Holland and Gallagher, 1993), and rats with lesions of the CA fail to show enhanced learning when the reward value is suddenly decreased, but they learn when the reward value unexpectedly increases (Holland, 2006). These results suggest that the CA is explicitly involved in negative PE signaling during appetitive learning. Similarly, several other animal studies also indicated that this structure encodes negative PEs in appetitive settings (Calu et al., 2010; Lee et al., 2010). Our results are consistent with these findings and show that bilateral CMA is the neural substrate of negative PE during appetitive learning in humans. As negative PEs lead to attenuation of both motivational significance and associative strength of stimuli (Rescorla and Wagner, 1972), the results indicate a role of the CMA in adjusting the behavioral response when the value of outcome decreases, as in the extinction process. Consistently, in rats, the CA updates reward expectancies during extinction learning Iordanova et al., 2016).

The findings of preferential activity within the CMA during negative PE signaling have implications for its role in flexible adaptive behaviors. In animals, CA was shown to be implicated in overexpectation (Haney et al., 2010; Holland, 2016; Iordanova et al., 2016), a method used to generate a negative PE and reduce conditioned responding to a previously trained cue. In addition, the interaction between the amygdala and OFC seems to be crucial for this process (Pujara et al., 2022). Our results are in line with these findings and suggest that the CMA and functional coupling between the CMA and OFC contribute to adaptive behavior driven by negative PEs.

Our results demonstrate that the right CMA is activated and functionally interacts with the OFC only during negative appetitive PE signaling. We are not aware of research that shows the involvement of the right amygdala in negative PE processing in appetitive context and not in aversive context. Nevertheless, our results may reflect processes in which the right amygdala has been suggested to play a role. For example, learning from a negative PE in the appetitive domain is closely associated with susceptibility to addiction. Addictive behaviors are related to the abnormal attribution of motivational significance to drug-associated cues and are resistant to extinction. Several studies point to the role of the right amygdala and prefrontal cortex in these processes. Functional connectivity analysis revealed that individuals who strongly overuse alcohol (Crane et al., 2018) and nicotine (Shen et al., 2017; Lin et al., 2021) have decreased connectivity between the right amygdala and OFC. Failing to learn from negative PE is observed in alcohol (Park et al., 2010) and nicotine (Chiu et al., 2008) addiction, and a deficiency in using negative PEs to adjust subsequent behavior is considered a central mechanism underlying addictive behaviors (Mathar et al., 2017). Thus, our findings may suggest that the right CMA is particularly involved in regulating behaviors related to reward seeking and, probably, to susceptibility to addiction.

Our results show that only the left CMA increases activity during aversive learning. Most studies on PE-related activity of the human amygdala have focused on fear learning and reported increased activity in either the BLA (Michely et al., 2020) or the CMA (Boll et al., 2013) subregions. However, the modality of stimuli used in these studies was different from our protocol. No studies have directly linked PE signaling in gustatory learning with the BLA and CMA subdivisions. However, when pavlovian learning involved an aversive gustatory stimulus (salty tea), the BOLD response in the left CMA correlated with the expected value signal (Prévost et al., 2013). This finding highlights the role of the left CMA in aversive gustatory learning, which is consistent with our results.

In addition, we explored the effects of personality traits of neuroticism and extraversion, as well as BMI, on the CMA and BLA activity. Our results did not confirm any predictions regarding appetitive PE signaling. We found that activation of the left CMA during the negative aversive PE was correlated with personality traits, that is, negatively correlated with neuroticism, and positively correlated with extraversion. Consistent with this result, previous studies revealed the modulatory impact of neuroticism on left amygdala activity during fear learning (Hooker et al., 2008). In addition, structural MRI data showed that neuroticism scores were correlated with the volume of the left amygdala (Koelsch et al., 2013). The positive correlation between extraversion and the aversive PE we observed agrees with previous results, showing a positive correlation between extraversion and fear extinction, which is a specific case of learning driven by negative aversive PEs (Rauch et al., 2005). Our data extend the previous findings by implicating the left CMA in this relationship. Together, the results suggest that weaker responses of the left CMA in more neurotic subjects may represent the maintained motivational significance of aversive stimuli when they are no longer relevant. In contrast, higher activation of the left CMA in more extroverted subjects may facilitate updating of the motivational significance of aversive stimuli when contingencies change.

Our results also showed that neither neuroticism nor extraversion correlated with task performance. This observation could be an effect of a relatively small sample size, which did not allow for covering a whole range of extraversion and neuroticism scores. On the other hand, although several conditioning studies showed clear effects on both behavioral and neural levels, significant correlations of neuroticism or extraversion were sometimes found only with BOLD responses, whereas this was not the case for behavioral performance. This dissociation has been observed in appetitive (Schweckendiek et al., 2016) and aversive conditioning (Lau et al., 2011) studies. It has been argued that subjective responses may be too insensitive to mirror individual differences (Schweckendiek et al., 2016). Future studies should explore this issue in more detail.

Disturbed eating behaviors have been linked to changed sensitivity to PE (Mathar et al., 2017). The response to PEs is also a potential neurobiological marker of eating disorder severity that can indicate individual treatment needs (DeGuzman et al., 2017). As our data indicate CMA involvement in negative PE signaling in gustatory associative learning, further investigation of its role in eating disorders appears essential.
